# Multiple benign metastasizing leiomyoma in the abdominal wall: a case report and literature review

**DOI:** 10.3389/fonc.2024.1391850

**Published:** 2024-05-17

**Authors:** Jiaqi Hu, Shuyuan Tian, Qing Pan, Yonghong Yu

**Affiliations:** ^1^ Department of Ultrasound Medicine, Tongde Hospital of Zhejiang Province, Hangzhou, China; ^2^ Department of Pathology, Tongde Hospital of Zhejiang Province, Hangzhou, China

**Keywords:** benign metastasizing leiomyoma, myomectomy, abdominal wall, smooth muscle cell, CEUS

## Abstract

Benign metastasizing leiomyoma (BML) is a rare disease that results from metastasis of uterine leiomyoma to distant sites with benign pathologic features. The lung is the most common metastatic site for BML. This report describes the case of a 49-year-old woman who presented with a mass in the abdominal wall with a surgical history of uterine myomectomy. Ultrasound and Magnetic resonance imaging (MRI) revealed multiple mass lesions. The histopathology of the mass specimen indicated BML. The imaging and clinical features of BML are discussed based on the characteristics of this case and related literature reports.

## Introduction

BML is a rare disease, first described by Steiner in 1939 (1). Although the disease appears benign in histomorphology, the biological behavior of the tumor is invasive. The most commonly involved organ is the lung, and others include lymph nodes, heart, skeletal muscle and pelvic cavity (2–4). BML is usually associated with a history of hysterectomy or myomectomy (5). A patient with BML in the abdominal wall is described in this study.

## Case presentation

A 49-year-old woman, gravida 1 para 1, accidentally presented with an egg-sized abdominal mass. Symptoms of fever, abdominal pain, and weight loss were not present, and she had previously undergone myomectomy. Physical examination revealed a rigid mass, with moderate motion and no tenderness.

**Figure 1:**
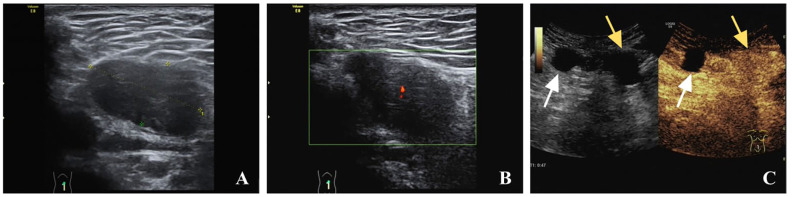
Abdominal ultrasonography, contrast-enhanced ultrasound (CEUS) of case. **(A)** Gray scale ultrasound showed a hypoechoic mass in the abdominal wall; **(B)** color Doppler flow imaging (CDFI) showed the mass with little blood flow signal; **(C)** CEUS showed hyper-enhancement of the large mass (yellow arrow) and no enhancement of the small mass (white arrow) in the arterial stage.

**Figure 2:**
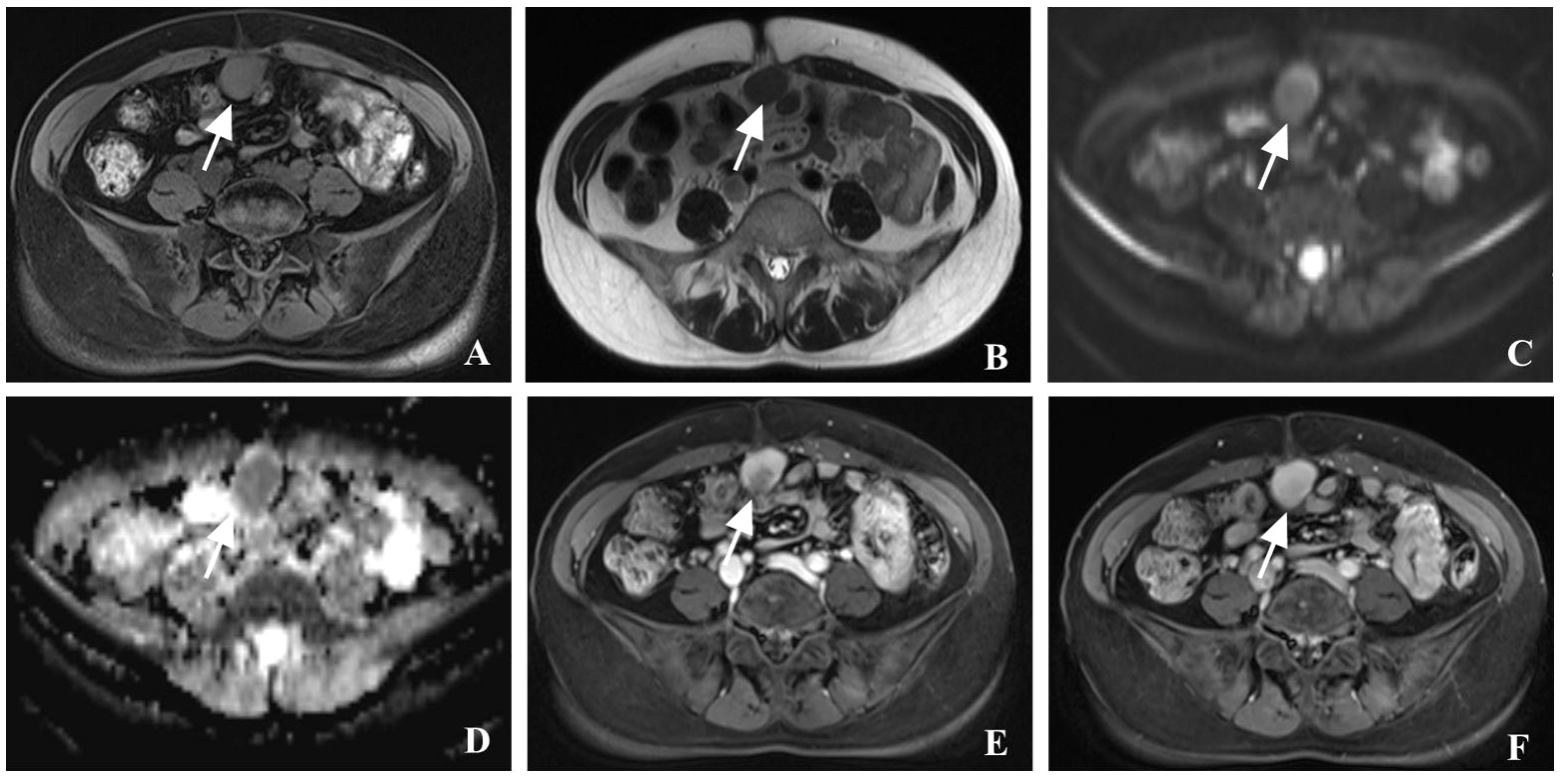
Contrast-enhanced magnetic resonance imaging (CE-MRI) of case. **(A–D)** MRI showed a slightly high signal in T1-weighted imaging, and a slightly high signal in T2-weighted imaging, diffusion-weighted imaging (DWI) and apparent diffusion coefficient (ADC); **(E, F)** CE-MRI showed that the mass progressive enhancement in the arterial phase and the delayed phase (see arrows).

Ultrasound examination showed multiple hypoechoic masses in the subcutaneous fascia layer. The two larger ones are approximately 41 x 25mm and 20 x 16mm with clear boundaries that were connected (**Figure 1A**). Color Doppler flow imaging (CDFI) showed a punctate blood flow signal within the mass (**Figure 1B**), and resistance index of 0.74. Contrast-enhanced ultrasound (CEUS) was performed using the contrast agent SonoVue. Simultaneously, an ultrasound-guided abdominal wall mass core needle biopsy was performed on the two masses. The larger mass showed relatively high enhancement in both arterial and venous phases. The smaller mass showed relatively no enhancement in the arterial phase and low enhancement around in venous phase (**Figure 1C**). An MRI was performed, which showed similar circular abnormal signal shadows on the anterior abdominal wall and abdominal cavity, with sizes of 29 x 16mm and 31 x 21mm, a slightly longer T1 and slightly longer T2, limited diffusion, and progressive enhancement after enhancement (**Figures 2A–F**). Chest Computed Tomography (CT) examination showed no abnormality. CEA, AFP, CA724, CA199 and CA125 markers were all normal.

Pathology of the biopsy suggested a spindle cell tumor, and combined with the patient’s surgical history, a fibromatosis in the abdominal wall was considered. The patient underwent the elective resection of the abdominal wall and abdominal cavity tumors. Two masses of 4 x 3cm and 2 x 2cm were observed during the operation, the larger one protruding into the abdominal cavity and the smaller one located in the anterior sheath of the rectus muscle. Two masses, about 2/3 of the rectus abdominis muscle and part of the posterior sheath, were removed completely. The surgeon performed tension-free repair of an abdominal wall hernia, inserting the patch into the abdominal cavity under the incision, fixing it on the abdominal wall, and suturing the rectus abdominis muscle and anterior sheath of the rectus abdominis muscle.

**Figure 3:**
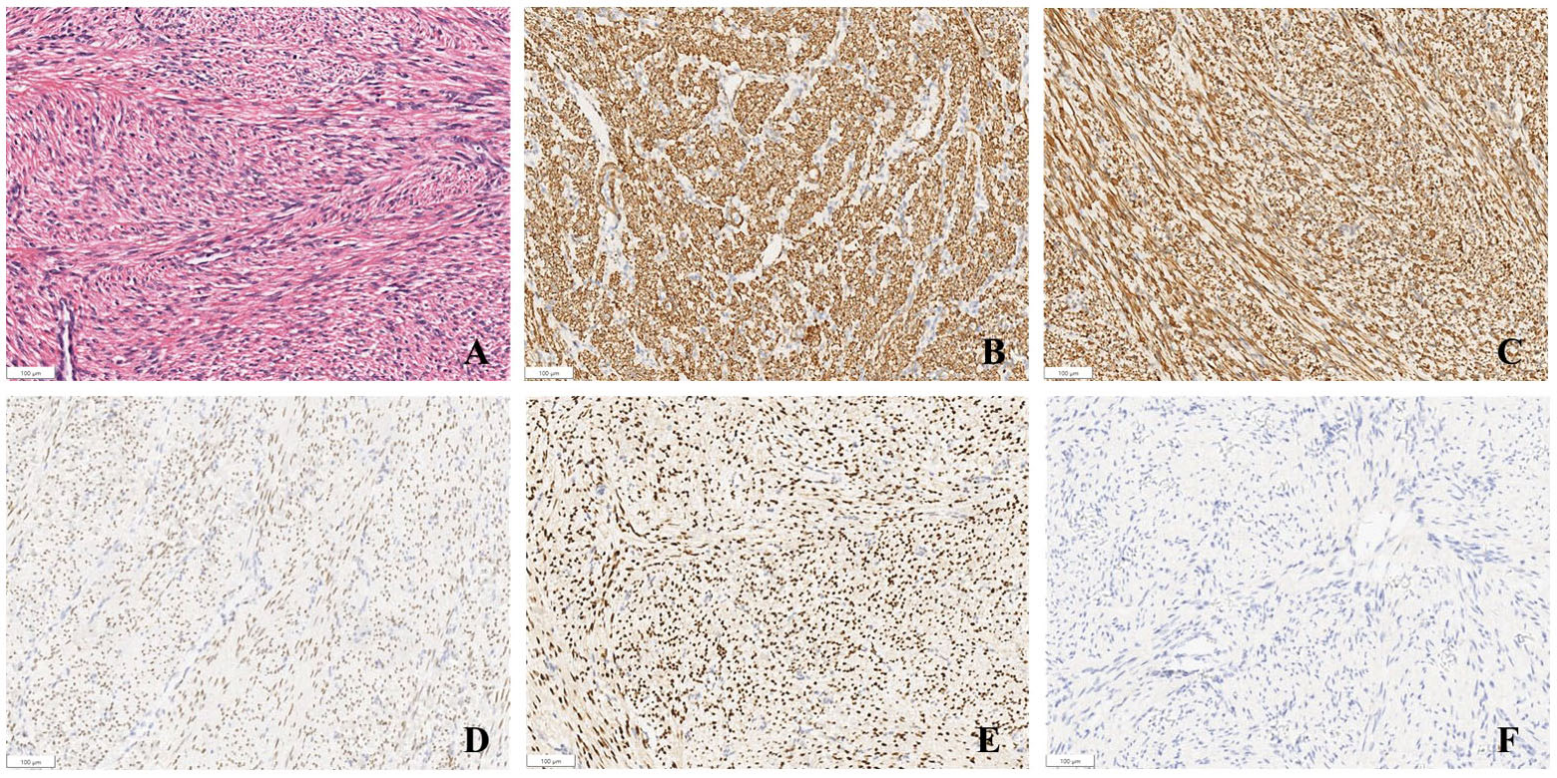
Pathology of case. **(A)** mass stained with hematoxylin and eosin (original magnification x 10); **(B–F)** SMA (+), DES (+), PR (+), ER (+), Ki-67 (<1%) was detected by immunohistochemical staining (original magnification x 10).

The histopathological examination showed spindle cells. Immunohistochemical staining showed that smooth muscle actin (SMA), desmin, estrogen receptor (ER) and progesterone receptor (PR) were positive, and cell proliferation antigen Ki-67 was less than 1% (**Figures 3A–F**). Based on these findings, the tumor was diagnosed as BML.

## Discussion

The mechanism of BML has been reported in various reports and remains controversial. Most instances of BML occur after uterine myomectomy or hysterectomy. The mechanism may be that surgery increases surgically induced hematogenous spread. In this case, iatrogenic peritoneal seeding theory seems to be a more reasonable explanation for the occurrence of BML (6, 7). Immunohistochemistry showed that ER and PR of tumor cells were positive, suggesting that BML may also be induced by hormone stimulation. Chromosomal abnormalities such as 19q, 22q, 1p, 13q deletion and 6p21 rearrangement can be found in these tumors (8). Other possible mechanisms include mesothelial metaplasia and misdiagnosed low-grade uterine leiomyosarcoma metastasis (9).

The clinical symptoms of BML are related to the affected sites. The most common site of metastasis is the lung, and the patients have no obvious clinical manifestations in the early stage, with a few having cough, chest pain, dyspnea and other symptoms (10, 11). In patients with BML involving the spine, leg pain and paresthesia have been reported (5). Patients with metastasis to the femur and muscles may have difficulty walking (12). BML involving the uterus, heart, and lymph nodes may be asymptomatic. Multiple abdominal wall metastases are rare, as reported in this case. BML with abdominal wall metastases has been reported in the literature (**Table 1**). Although most cases of BML occur following myomectomy or hysterectomy, some cases of BML are not related to prior surgical history (22). Studies have shown that BML can occur in premenopausal or postmenopausal women, and up to 30% of cases may have respiratory symptoms (23).

**Table 1:** Summary of BML cases with abdominal wall metastasis.

Metastasis	Age	History of gynecologic surgery	Surgical treatment
abdominal wall and pelvic cavity	37 (13)	myomectomy 10 years earlier	extirpation of the abdominal and pelvic tumors and partial greater omentum
abdominal wall and pelvic cavity	46 (14)	hysterectomy 8 years earlier	extirpation of the abdominal and pelvic tumors and bilateral oophorectomy
abdominal wall and pulmonary	33 (15)	myomectomy 13 years earlier	extirpation partial tumors
abdominal wall	42 (16)	mysterectomy 8 years earlier	extirpation of the tumors
abdominal wall	44 (17)	hysterectomy	extirpation of the tumors
abdominal wall and pulmonary	44 (18)	myomectomy	N/A
abdominal wall and pelvic cavity	49 (18)	myomectomy 12 years earlier	N/A
abdominal wall, pelvic cavity and pulmonary	47 (19)	twice myomectomy and hysterectomy	N/A
abdominal wall and pulmonary	44 (20)	myomectomy and abdominal hysterectomy	extirpation of the tumors in pulmonary
multiple metastasis (abdominal wall, ventricle wall, lungs, liver, muscles, and pelvic cavity)	37 (21)	twice myomectomy	hysterectomy and resection of the nodules in the mesosalpinx, mesenterium, and abdominal wall
abdominal wall, abdominal and pelvic cavity	54 (9)	abdominal hysterectomy and bilateral salpingo-oophorectomy 6 years earlier	extirpation of the tumors

BML is radiographically characterized as a benign mass. On ultrasound, BML can be manifested as multiple hypoechoic masses in the abdominal wall or pelvic cavity, and blood flow signals can be seen inside. It can be shown as multiple nodules with clear boundaries on CT, with no significant enhancement on enhanced CT (24). Notably, fluorodeoxyglucose (FDG) -positron emission tomography (PET)/CT showed that FDG uptake by tumors was all within the physiological range (12). According to the review of the FDG-PET/CT examination results of BML, the FDG uptake level of more than 90% of BML patients is lower than the medium level (25), which can be used as one of the crucial bases for the differentiation of benign and malignant tumors. Therefore, physicians must maintain heightened awareness and index of suspicion when approaching a woman with a mass in any region of the abdomen or pelvis. Further investigation with abdominal and pelvic ultrasonography and magnetic resonance imaging or computed tomography is necessary. Benign lesions can be found even in patients presenting with giant masses and higher CA125 than normal levels (26). CA125 and CA199 are considered to be important gynecological tumor indicators, which are commonly seen in malignant tumor diseases, and their elevated levels do not represent absolute malignancy (27, 28).

Regarding pathological diagnostic criteria for BML, BML presents as an extra-uterine smooth muscle cell proliferation with histological features similar to those of primary benign uterine fibroids. Uterine leiomyoblastoma can also exhibit some malignant features, making it difficult for physicians to distinguish (29). Immunohistochemistry is also very helpful in differentiating leiomyoma from leiomyosarcoma. Studies have shown that the positive rate of ER/PR in MBL patients is over 90%, and only 4.4% are negative at the same time (30). ER/PR positivity is specific for uterine leiomyomas and may be helpful in diagnosing BML. In the present case, the abdominal mass was excised, and immunohistochemistry confirmed that the patient’s ER/PR was positive.

There is currently no standardized treatment for BML. Due to the behavioral characteristics of the slow growth of BML, most lesions do not change in size after long-term follow-up, and regular follow-up and periodic imaging examinations can be chosen. Since BML can be hormone-dependent (ER and PR-positive), hormone therapy such as GnRH-a, progesterone antagonists (Mifepristone), selective estrogen receptor modulators (SERMs, tamoxifen) and aromatase inhibitors may be considered, or even surgery to remove the ovaries, particularly for patients with symptomatic lung involvement (24, 30, 31). Although bilateral oophorectomy is commonly used, unilateral oophorectomy is sufficient to promote the reduction of lung BML mass (2). In addition, surgical removal of the tumor is an effective treatment.

## Conclusion

The characteristics of low specificity and incidence rate of BML can lead physicians to be unfamiliar with the disease, enabling it to be misdiagnosed as a malignant tumor. Asking operation history of myomectomy or hysterectomy can help us diagnose BML. Pathological diagnosis plays an essential role in determining the follow-up treatment. Obtaining part of the specimens through ultrasound-guided tissue core needle biopsy is effective and safe and can be used as one of the recommended methods.

## Data availability statement

The original contributions presented in the study are included in the article/supplementary material. Further inquiries can be directed to the corresponding author.

## Ethics statement

Written informed consent was obtained from the individual(s) for the publication of any potentially identifiable images or data included in this article.

## Author contributions

JH: Writing – original draft. ST: Writing – review & editing. QP: Writing – review & editing. YY: Writing – review & editing.
